# Supplementary material for: onEEGwaveLAD: A fully automated online EEG wavelet-based learning adaptive denoiser for artefacts identification and mitigation
Source: PLoS One. 2025 Jan 28;20(1):e0313076. doi: 10.1371/journal.pone.0313076 (PMC11774379; doi:10.1371/journal.pone.0313076)
Supplement: S1 Appendix — (PDF) [file pone.0313076.s001.pdf]

# 7 Appendix

## 7.1 Algorithm for offline blink selection

**Algorithm 2** Plausible blinks selection (Ground truth formation)

$patience \leftarrow 6$
$noImprovement \leftarrow 0$
$blinksCount \leftarrow 0$
**for** $k$ `in range(70,1000, 30)` **do**
    $[trueBlinks] \leftarrow []$
    $[peaks] \leftarrow PeakDetection([VEOGsig], k)$
    **for** $p$ `in` $[peaks]$ **do**
        $[CH_{recording}] \leftarrow getChannelsRecordingAtPeak(p)$
        $[OCH_{recording}] \leftarrow orderByHighest(CH_{recordings})$
        $[CH_{nm}] \leftarrow getChannelsNames(OCHs_{recordings})$

        $[cLocsPF] \leftarrow [Fp1, Fp2]$ ▷ Cornea locations in PF
        $[cLocsP] \leftarrow [F7, F3, Fz, F4, F8]$ ▷ Cornea locations in F
        $[rLocsO] \leftarrow [O1, O2, Oz]$ ▷ Retina locations in O
        $[rLocsPO] \leftarrow [PO7, PO3, P9, PO4, PO8, P10]$ ▷ Retina locations in PO/P
        $[rLocs] \leftarrow [retinaLocsO] + [retinaLocsPO]$

        $[intPeakFp1] \leftarrow getInterval(Fp1, p, 512)$ ▷ 512 values +-peak of Fp1
        $[intPeakFp2] \leftarrow getInterval(Fp2, p, 512)$ ▷ 512 values +-peak of Fp2
        $[zFp1] \leftarrow z([intPeakFp1])$ ▷ Compute z-scores of interval
        $[zFp2] \leftarrow z([intPeakFp1])$ ▷ Compute z-scores of interval
        $Fp1PeakVal \leftarrow [intPeakFp1][512]$ ▷ get peak value of Fp1
        $Fp2PeakVal \leftarrow [intPeakFp2][512]$ ▷ get peak value of Fp2
        $std_{th} \leftarrow 2$ ▷ Std threshold

        **if** $[CH_{nm}]$[0] in cLocsPF AND $[CH_{nm}]$[1] in cLocsPF
         AND $[CH_{nm}]$[2] in cLocsF) AND $[CH_{nm}]$[3] in cLocsF
         AND $[CH_{nm}]$[-1] in rLocs AND $[CH_{nm}]$[-2] in rLocs **then**
            **if** $zFp1$[512] >= $std_{th}$ AND $zFp2$[512] >= $std_{th}$ **then**
                $[trueBlinks] \leftarrow [trueBlinks] + p$ ▷ add true positive blink's location
            **end if**
        **end if**
    **end for**
    $noImprovement \leftarrow noImprovement + 1$
    **if** $len([trueBlinks]) < blinksCount$ **then**
        $noImprovement \leftarrow 0$
    **end if**
    **if** $noImprovement = patience$ **then**
        exit()
    **end if**
**end for**

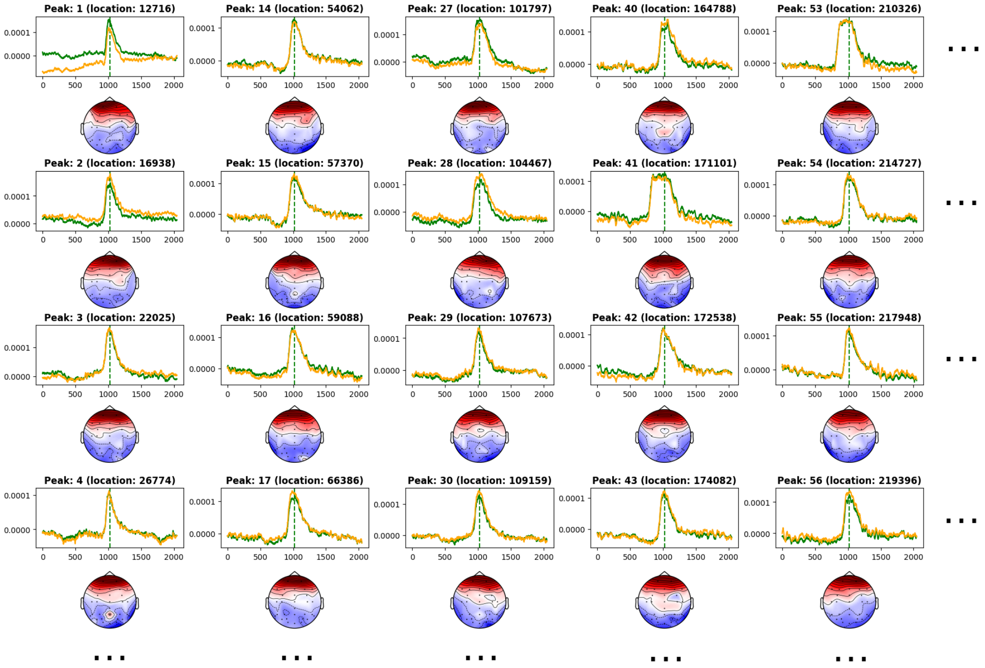


**Fig 6.** An example of ground truth formation with valid blinks out of the many peaks identified by the offline blink detection algorithm for a single participant.

## 7.2 Signal-to-noise ratios

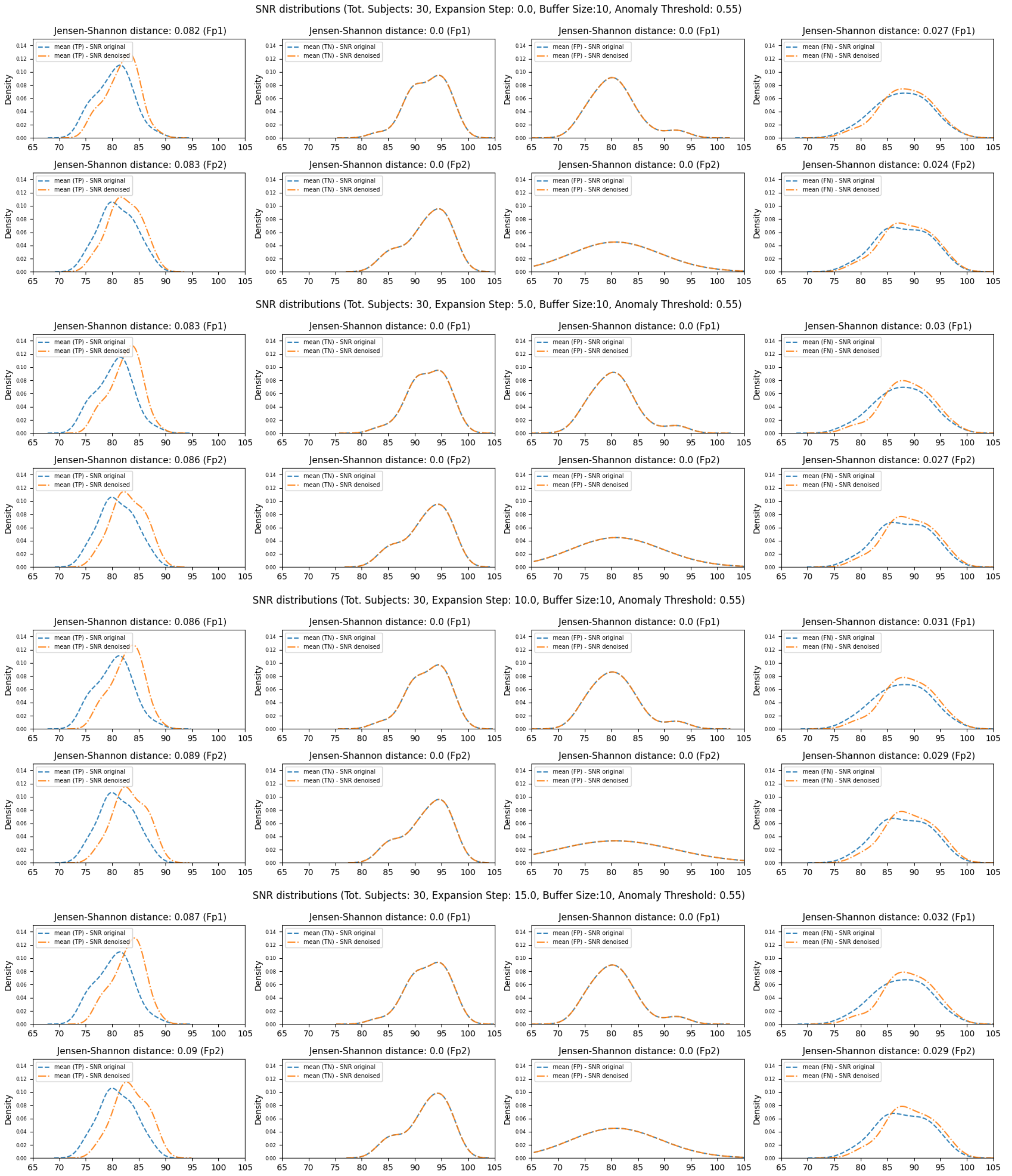


**Fig 7.** Probability distributions of the means of the signal-to-noise ratios (SNR) for all the EEG windows processed for all the subjects, grouped by prediction category (TP=True Positives, TN=True Negatives, FP=False Positives, FN=False Negatives) for a specific instantiation of the *onEEGwaveLAD* pipeline (EEG Window Length=1000ms, Sampling rate=1024, Mother wavelet=Sym4, Buffer capacity=10, IF sub-sampling size=512, Number of IF trees=100, Anomaly Threshold:0.55, Expansion step=[0, 5, 10, 15])

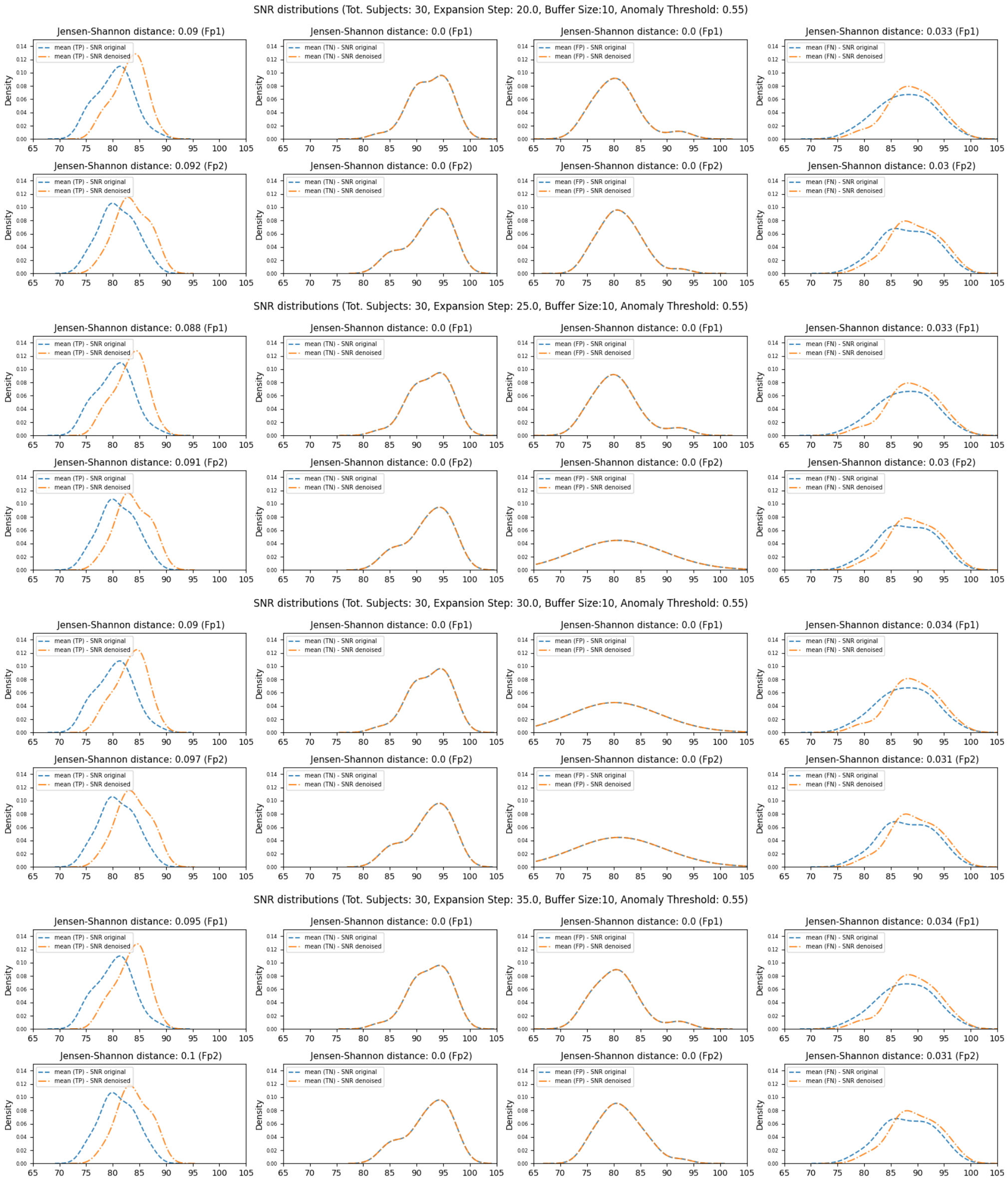


**Fig 8.** Distributions of the means of the signal-to-noise ratios (SNR) for all the EEG windows processed for all the subjects, grouped by prediction category (TP=True Positives, TN=True Negatives, FP=False Positives, FN=False Negatives) for a specific instantiation of the *onEEGwaveLAD* pipeline (EEG Window Length=1000ms, Sampling rate=1024, Mother wavelet=Sym4, Buffer capacity=10, IF sub-sampling size=512, Number of IF trees=100, Anomaly Threshold:0.55, Expansion step=[20, 25, 30, 35])

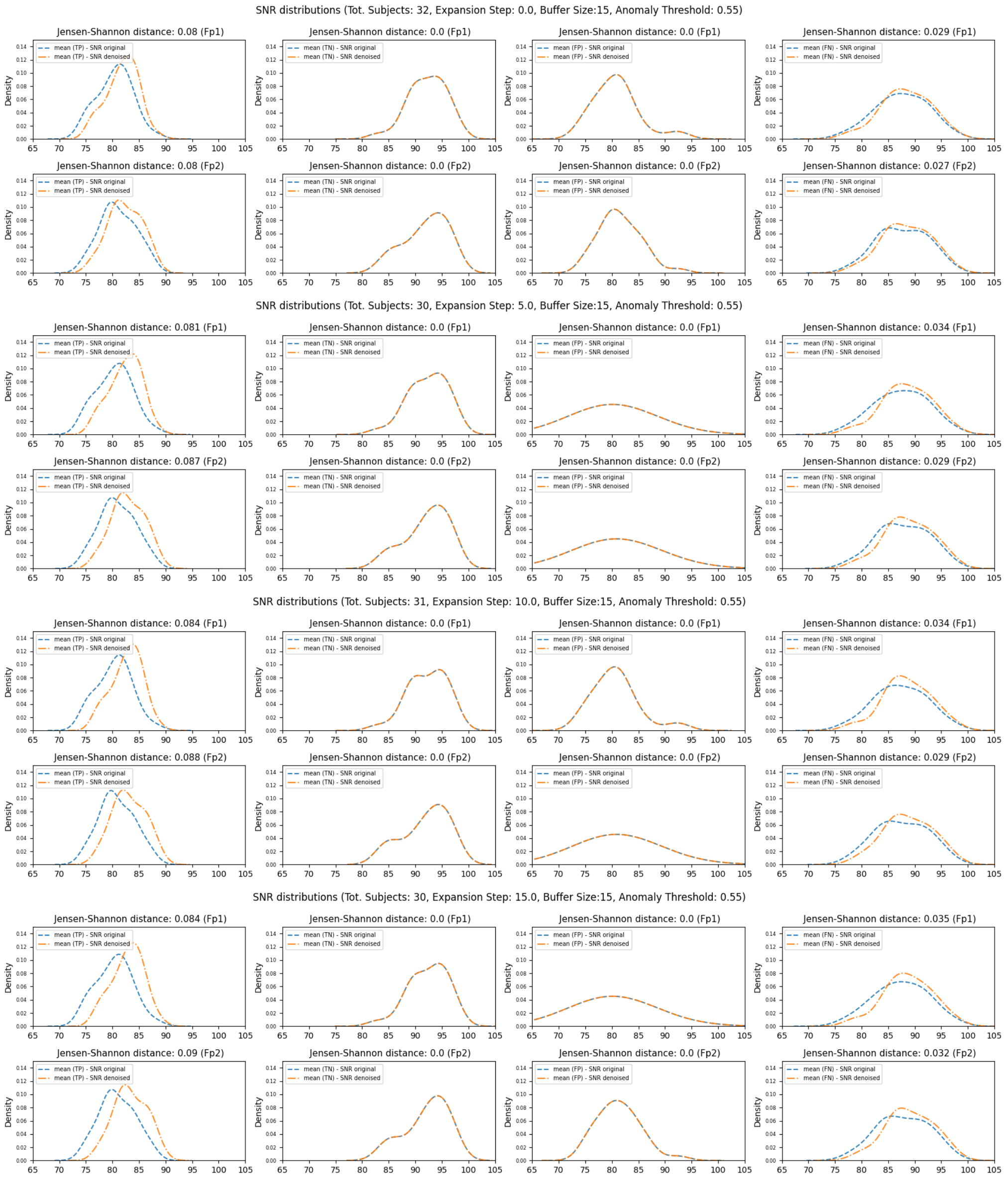


**Fig 9.** Probability distributions of the means of the signal-to-noise ratios (SNR) for all the EEG windows processed for all the subjects, grouped by prediction category (TP=True Positives, TN=True Negatives, FP=False Positives, FN=False Negatives) for a specific instantiation of the *onEEGwaveLAD* pipeline (EEG Window Length=1000ms, Sampling rate=1024, Mother wavelet=Sym4, Buffer capacity=15, IF sub-sampling size=512, Number of IF trees=100, Anomaly Threshold:0.55, Expansion step=[0, 5, 10, 15])

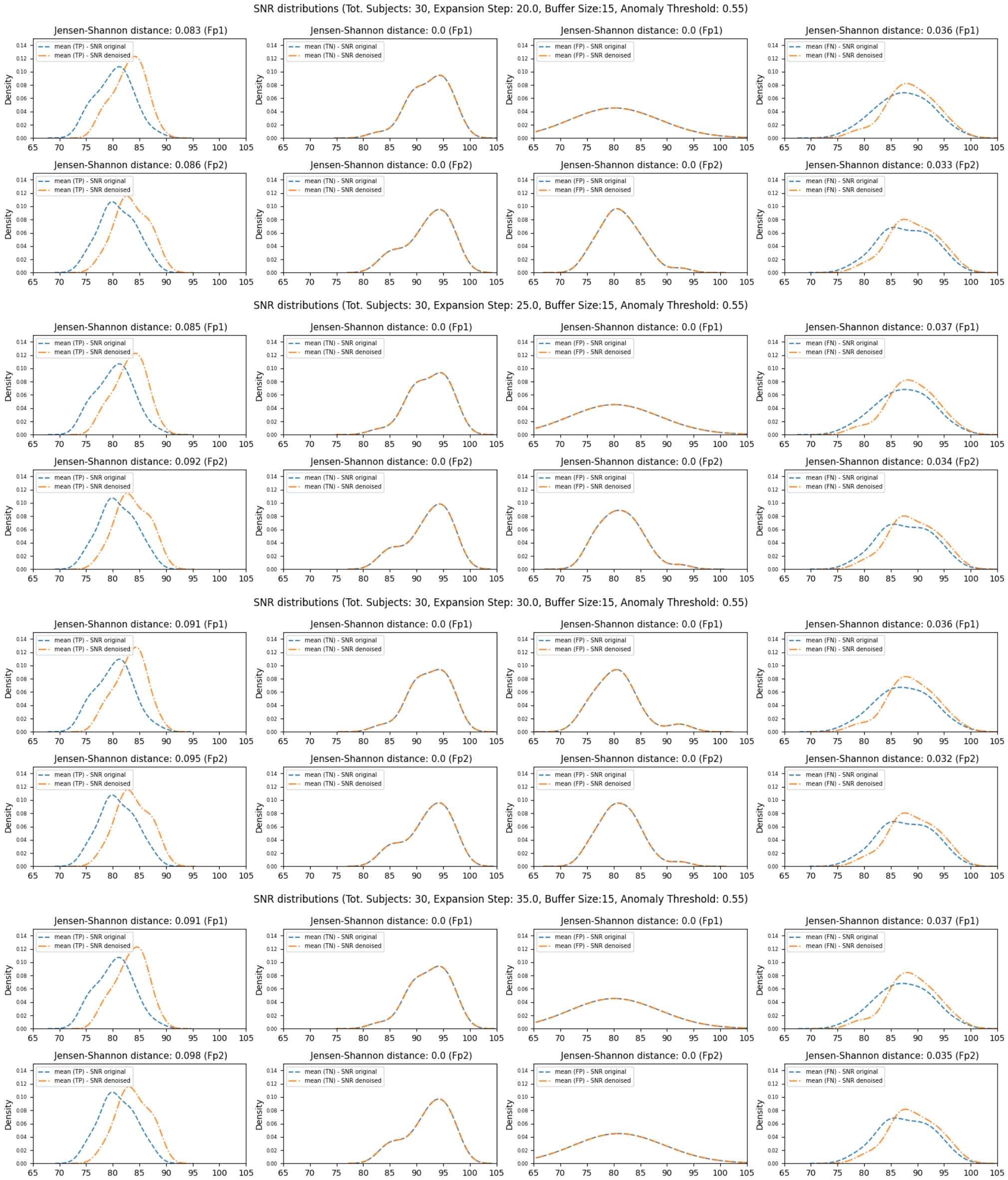


**Fig 10.** Probability distributions of the means of the signal-to-noise ratios (SNR) for all the EEG windows processed for all the subjects, grouped by prediction category (TP=True Positives, TN=True Negatives, FP=False Positives, FN=False Negatives) for a specific instantiation of the *onEEGwaveLAD* pipeline (EEG Window Length=1000ms, Sampling rate=1024, Mother wavelet=Sym4, Buffer capacity=15, IF sub-sampling size=512, Number of IF trees=100, Anomaly Threshold:0.55, Expansion step=[20, 25, 30, 35])

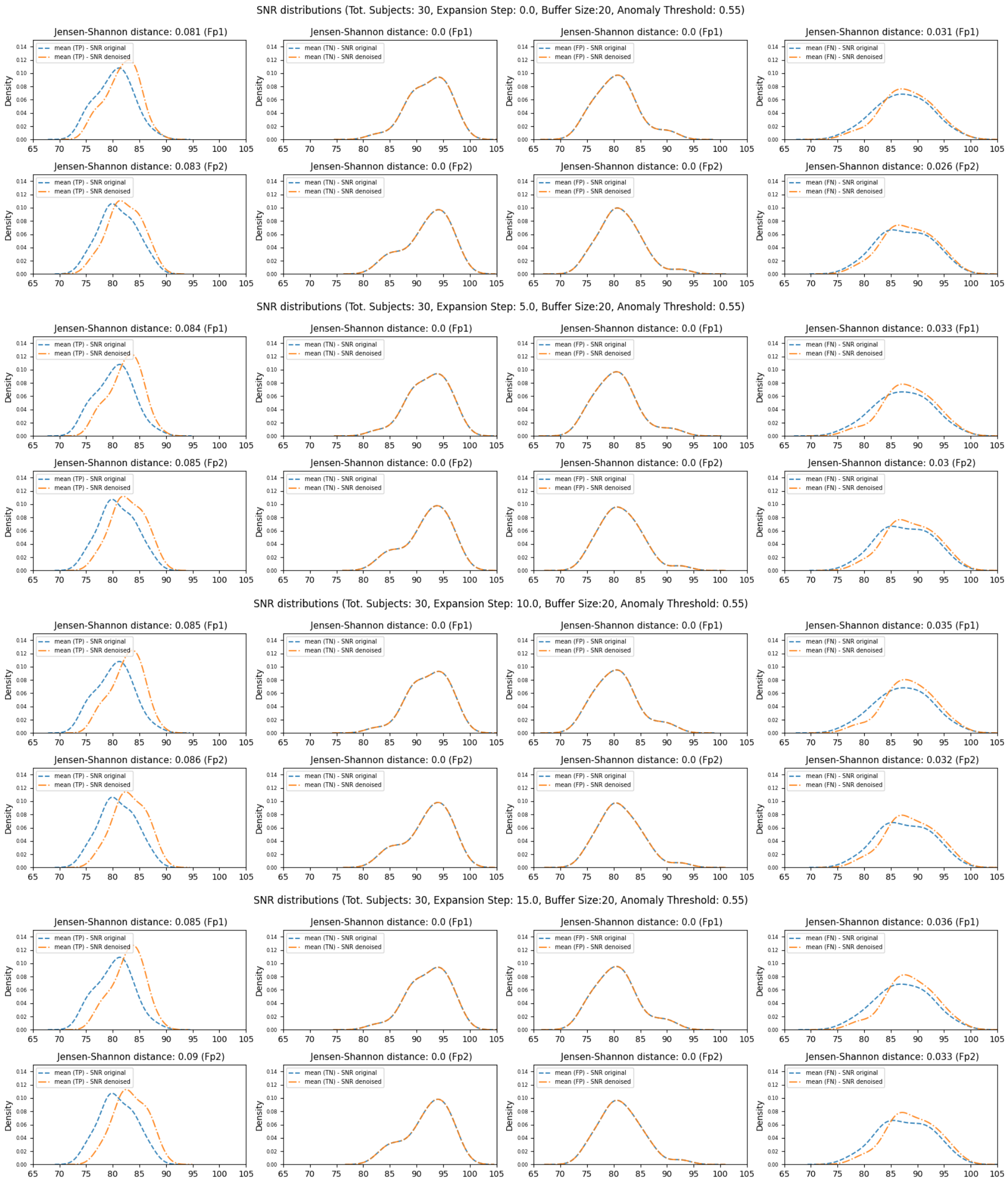


**Fig 11.** Probability distributions of the means of the signal-to-noise ratios (SNR) for all the EEG windows processed for all the subjects, grouped by prediction category (TP=True Positives, TN=True Negatives, FP=False Positives, FN=False Negatives) for a specific instantiation of the *onEEGwaveLAD* pipeline (EEG Window Length=1000ms, Sampling rate=1024, Mother wavelet=Sym4, Buffer capacity=20, IF sub-sampling size=512, Number of IF trees=100, Anomaly Threshold:0.55, Expansion step=[0, 5, 10, 15])

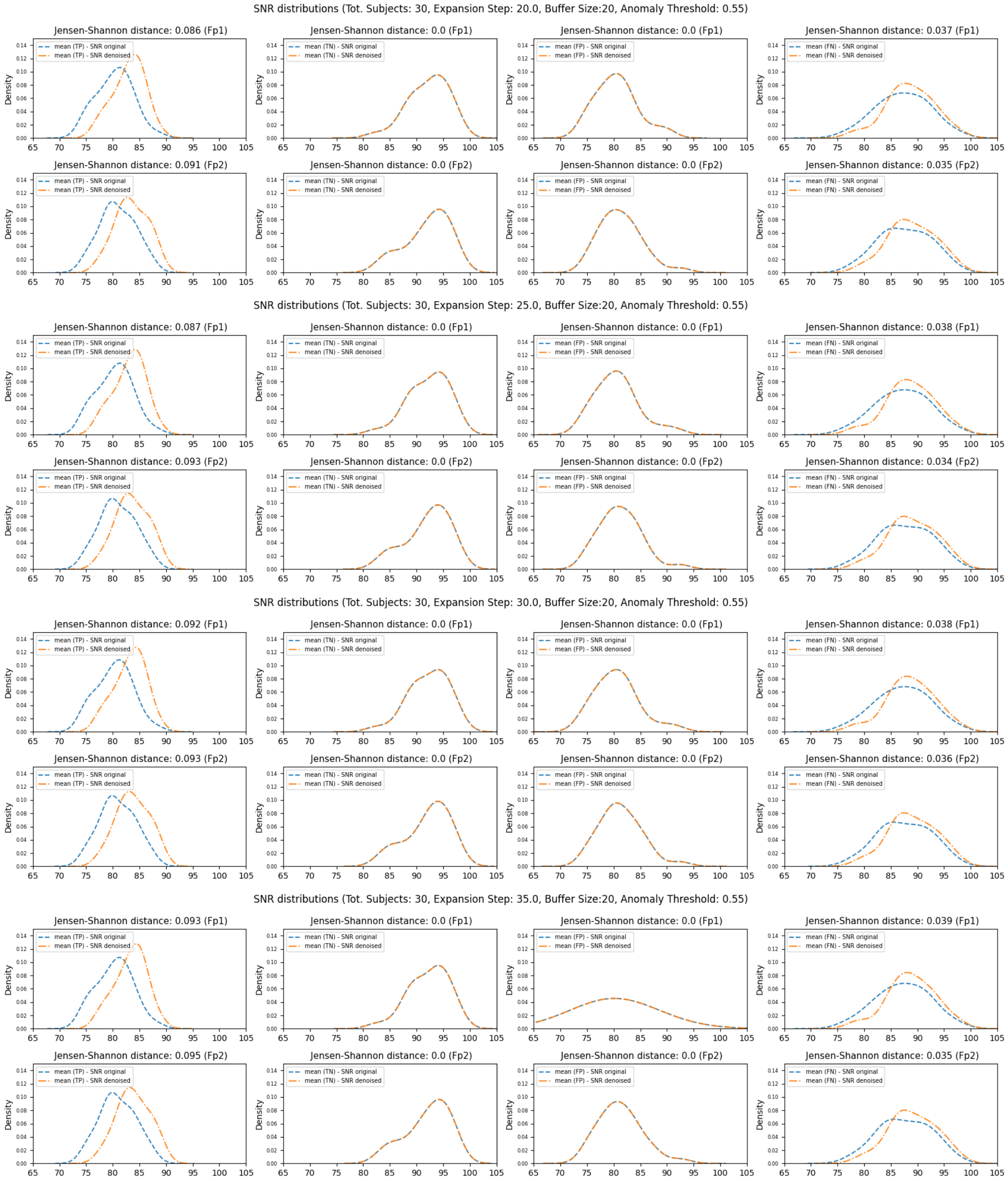


**Fig 12.** Probability distributions of the means of the signal-to-noise ratios (SNR) for all the EEG windows processed for all the subjects, grouped by prediction category (TP=True Positives, TN=True Negatives, FP=False Positives, FN=False Negatives) for a specific instantiation of the *onEEGwaveLAD* pipeline (EEG Window Length=1000ms, Sampling rate=1024, Mother wavelet=Sym4, Buffer capacity=20, IF sub-sampling size=512, Number of IF trees=100, Anomaly Threshold:0.55, Expansion step=[20, 25, 30, 35])

## 7.3 Examples of denoised EEG windows for true positives

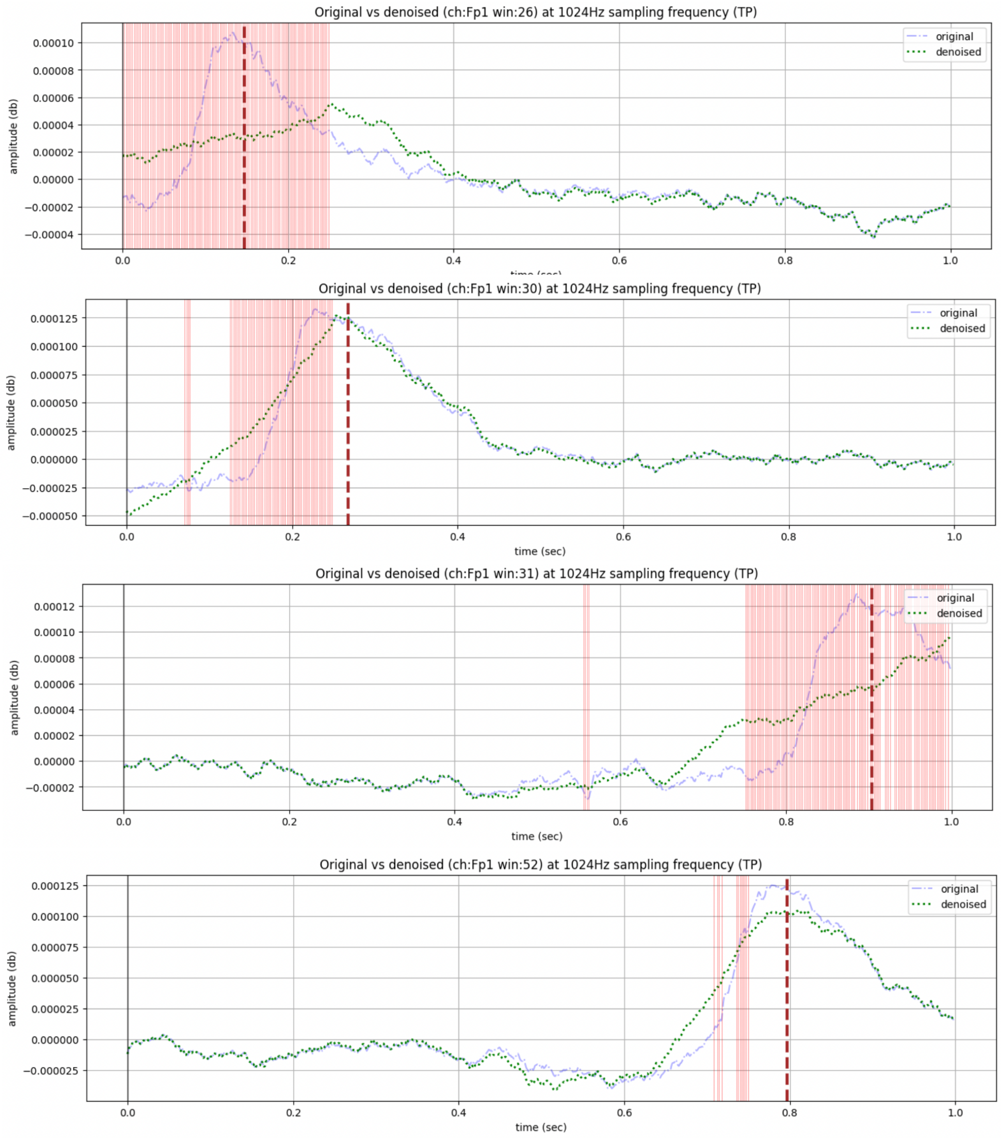


**Fig 13.** Examples of denoised EEG windows for true positives (part A)

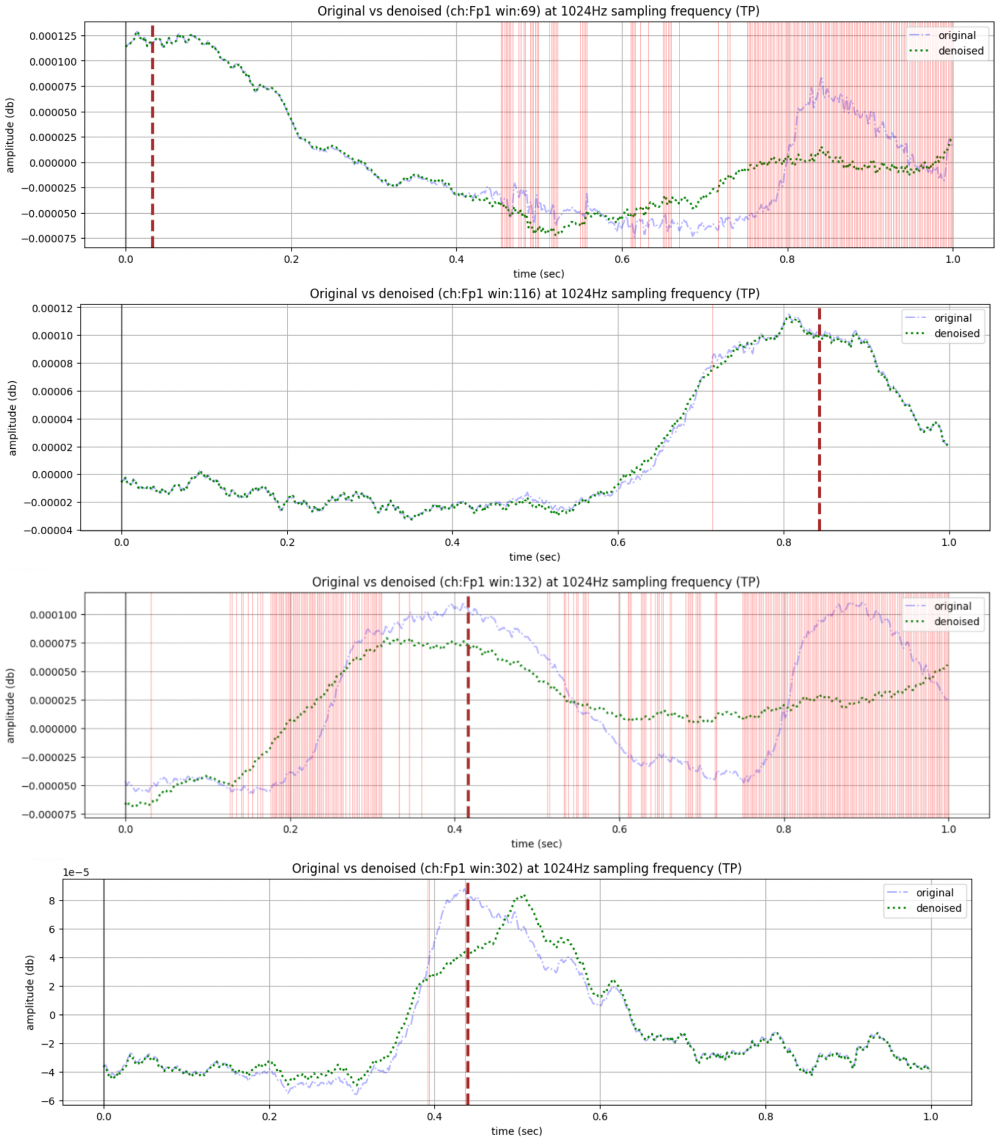


**Fig 14.** Examples of denoised EEG windows for true positives (part B)

## 7.4 Examples of denoised EEG windows for true negatives

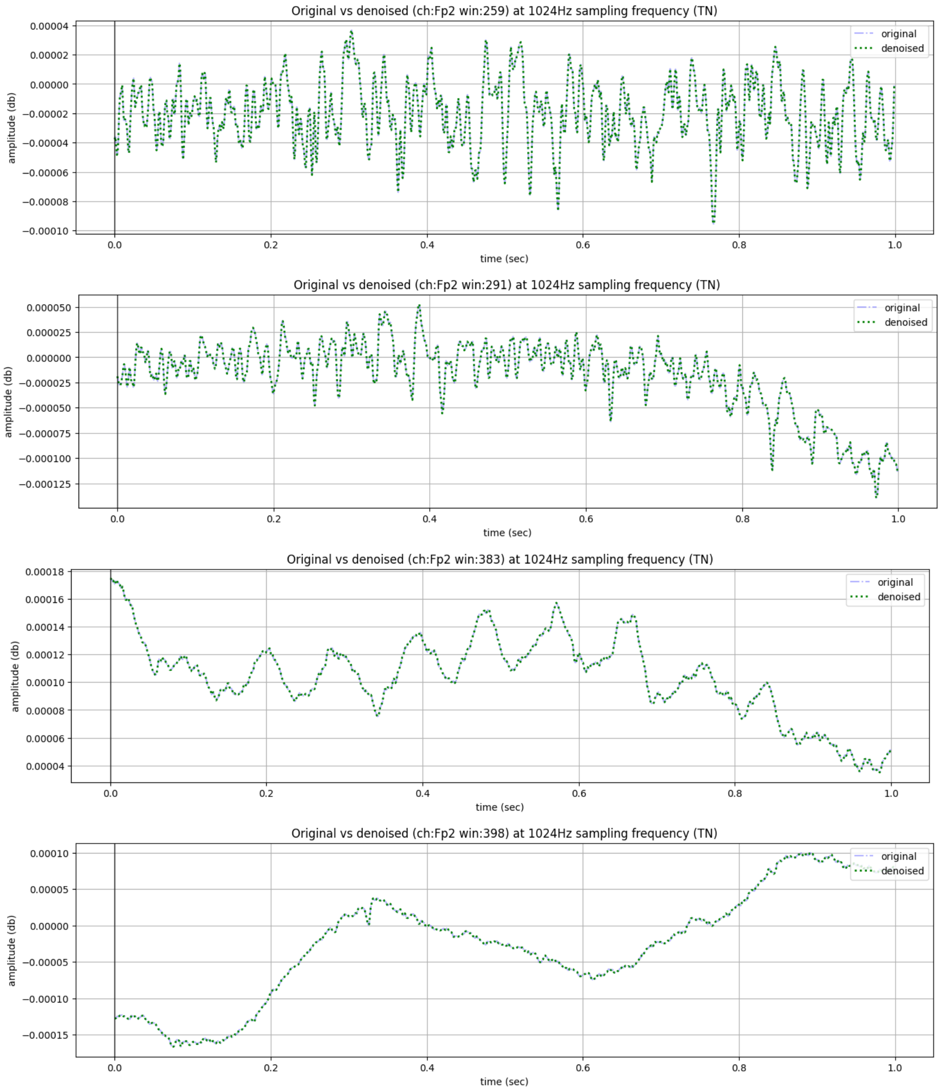


**Fig 15.** Examples of denoised EEG windows for true negatives

## 7.5 Examples of denoised EEG windows for false positives

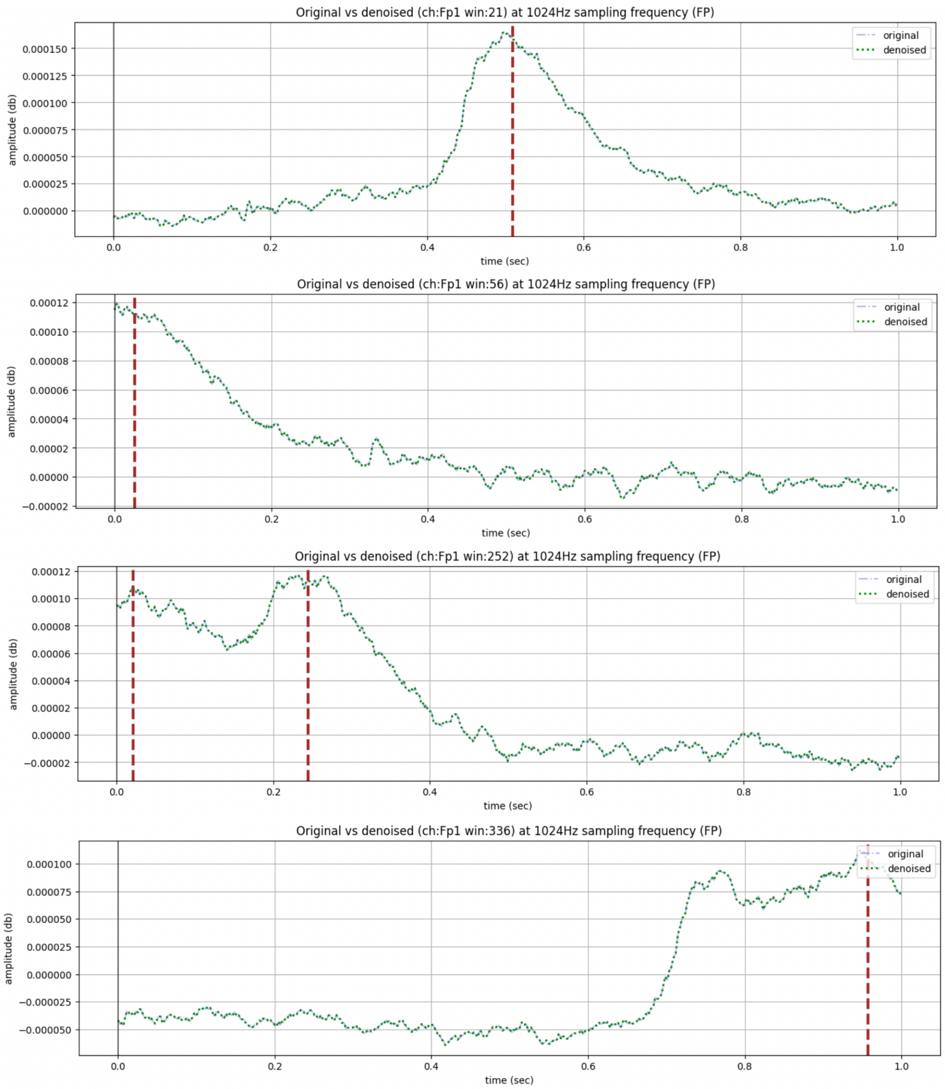


**Fig 16.** Examples of denoised EEG windows for false positives

## 7.6 Examples of denoised EEG windows for false negatives

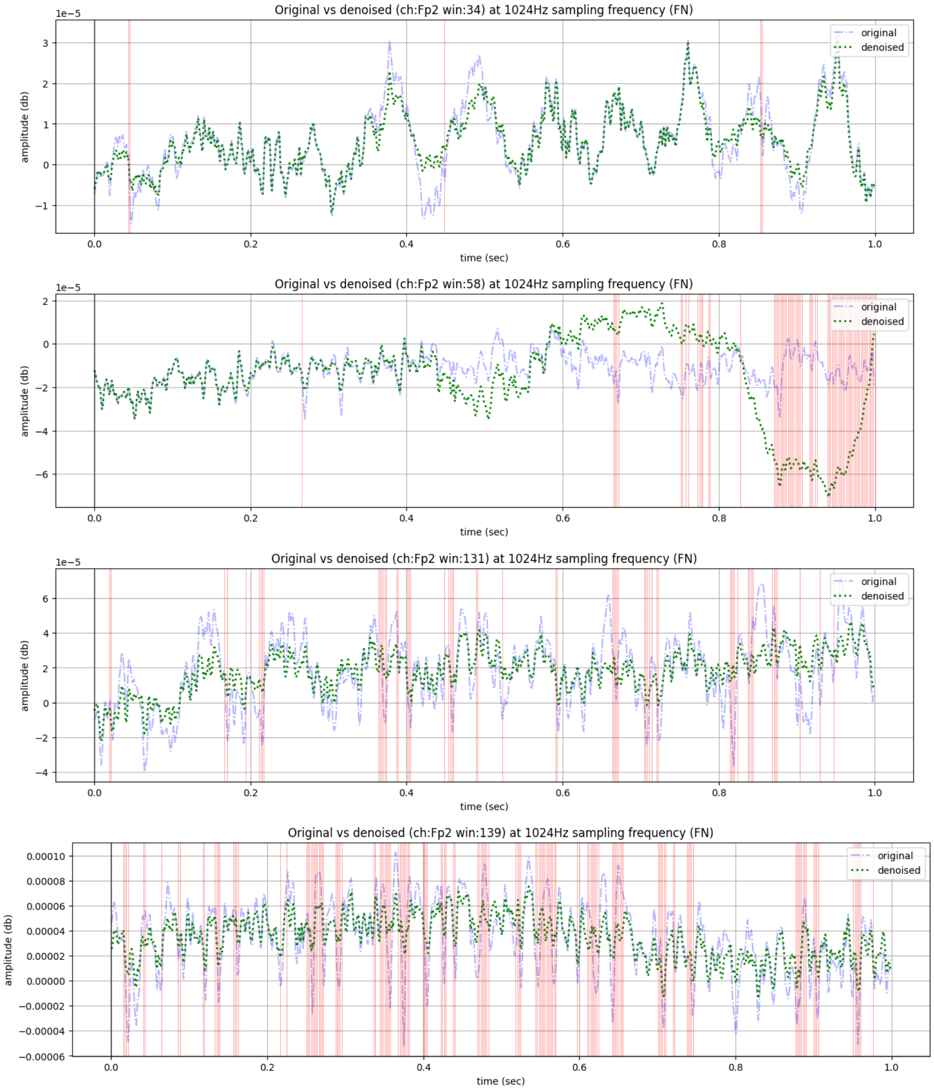


**Fig 17.** Examples of denoised EEG windows for false negatives (part A)

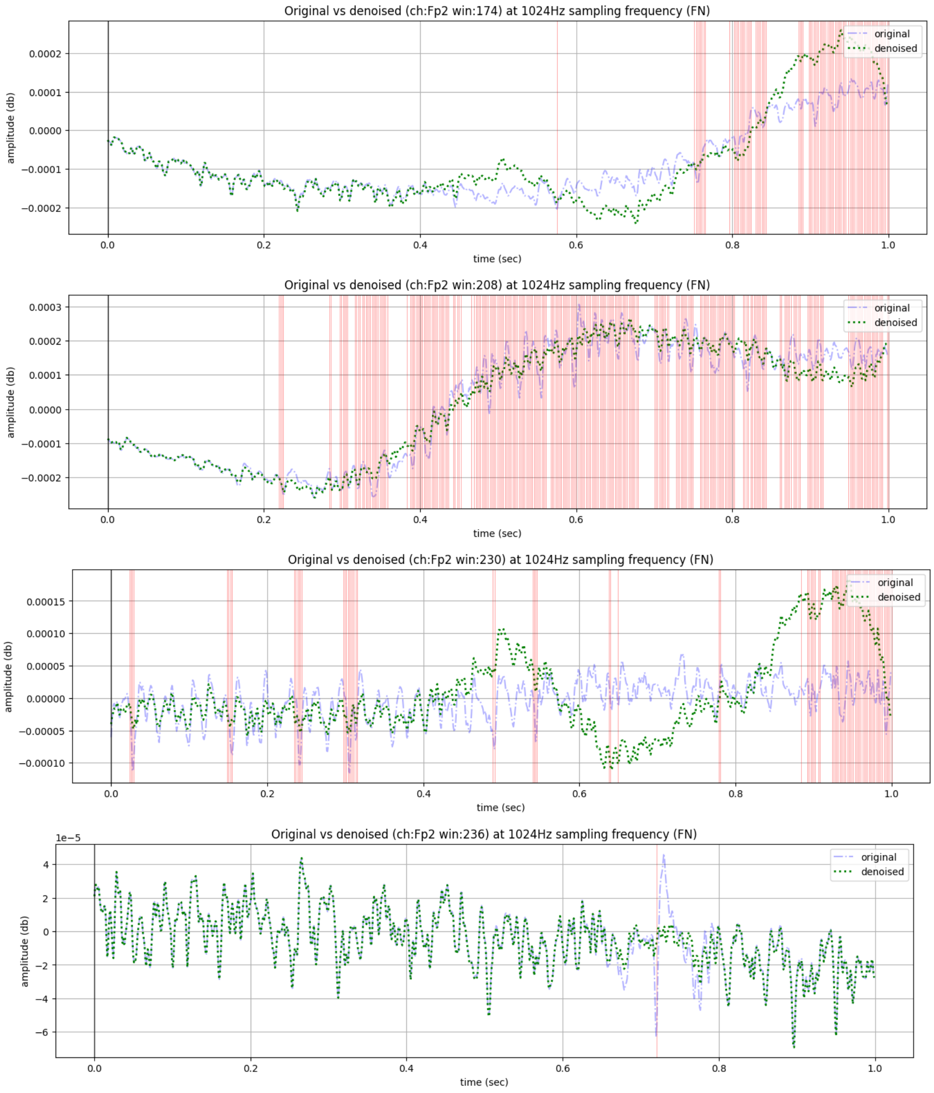


**Fig 18.** Examples of denoised EEG windows for false negatives (part B)
