# Supplementary material for: onEEGwaveLAD: A fully automated online EEG wavelet-based learning adaptive denoiser for artefacts identification and mitigation
Source: PLoS One. 2025 Jan 28;20(1):e0313076. doi: 10.1371/journal.pone.0313076 (PMC11774379; doi:10.1371/journal.pone.0313076)
Supplement: Attachment — Submitted filename: Reviewer Comments PONE-D-24-35626.docx [file pone.0313076.s002.docx]

**Reviewer Comments**

**onEEGwaveLAD: A fully automated online EEG wavelet-based Learning Adaptive Denoiser**

Q1. Why was sym4 mother wavelet chosen among other wavelets for noise reduction in EEG Signal?

Q2. What was the duration of the EEG data for the 37 participants from the N170 dataset of ERP CORE form and how does the other artefacts such as muscle movements impact the signal?

Q3. It is mentioned in the manuscript that the anomalies in this algorithm is assumption of specific data points in cardinality and different from other points-Justify

Q4. Does all the data from dataset is included as random dimension values rd1 to rdc ?

Q4. Figure 2,3 image is not clear , can use a HD image**.**
